# Hypoxia-inducible factor-2α mediates senescence-associated intrinsic mechanisms of age-related bone loss

**DOI:** 10.1038/s12276-021-00594-y

**Published:** 2021-04-02

**Authors:** Sun Young Lee, Ka Hyon Park, Gyuseok Lee, Su-Jin Kim, Won-Hyun Song, Seung-Hee Kwon, Jeong-Tae Koh, Yun Hyun Huh, Je-Hwang Ryu

**Affiliations:** 1 Department of Pharmacology and Dental Therapeutics, School of Dentistry, Chonnam National University, Gwangju, 61186 Republic of Korea; 2 Hard-tissue Biointerface Research Center, School of Dentistry, Chonnam National University, Gwangju, 61186 Republic of Korea; 3 School of Life Sciences, Gwangju Institute of Science and Technology (GIST), Gwangju, 61005 Republic of Korea

**Keywords:** Cell biology, Diseases

## Abstract

Aging is associated with cellular senescence followed by bone loss leading to bone fragility in humans. However, the regulators associated with cellular senescence in aged bones need to be identified. Hypoxia-inducible factor (HIF)−2α regulates bone remodeling via the differentiation of osteoblasts and osteoclasts. Here, we report that HIF-2α expression was highly upregulated in aged bones. HIF-2α depletion in male mice reversed age-induced bone loss, as evidenced by an increase in the number of osteoblasts and a decrease in the number of osteoclasts. In an in vitro model of doxorubicin-mediated senescence, the expression of *Hif-2*α and *p21*, a senescence marker gene, was enhanced, and osteoblastic differentiation of primary mouse calvarial preosteoblast cells was inhibited. Inhibition of senescence-induced upregulation of HIF-2α expression during matrix maturation, but not during the proliferation stage of osteoblast differentiation, reversed the age-related decrease in *Runx2* and *Ocn* expression. However, HIF-2α knockdown did not affect *p21* expression or senescence progression, indicating that HIF-2α expression upregulation in senescent osteoblasts may be a result of aging rather than a cause of cellular senescence. Osteoclasts are known to induce a senescent phenotype during in vitro osteoclastogenesis. Consistent with increased HIF-2α expression, the expression of *p16* and *p21* was upregulated during osteoclastogenesis of bone marrow macrophages. ChIP following overexpression or knockdown of HIF-2α using adenovirus revealed that *p16* and *p21* are direct targets of HIF-2α in osteoclasts. Osteoblast-specific (*Hif-2α*^fl/fl^;*Col1a1*-*Cre*) or osteoclast-specific (*Hif-2α*^fl/fl^;*Ctsk*-*Cre*) conditional knockout of HIF-2α in male mice reversed age-related bone loss. Collectively, our results suggest that HIF-2α acts as a senescence-related intrinsic factor in age-related dysfunction of bone homeostasis.

## Bone remodeling: Protein linked to age-related bone loss

Drugs directed at a protein called HIF-2α may help combat cellular aging processes in bone. Je-Hwang Ryu from Chonnam National University in Gwangju, South Korea, and colleagues have linked the expression of HIF-2α, a known regulator of bone remodeling, to aging and senescence, a state of irreversible growth arrest in which cells remain metabolically active yet cease dividing. Experimentally targeting HIF-2α in mice, either in bone-forming osteoblast cells or in bone-resorbing osteoclast cells, reversed the gradual and progressive decline of bone tissue associated with aging, showing that senescence-induced HIF-2α activity contributes to imbalances in bone cell turnover. HIF-2α blockers are already in clinical testing for the treatment of cancer; these findings suggest that this class of drugs should now be considered for the treatment of osteoporosis and other bone diseases as well.

## Introduction

Bone homeostasis is maintained by tight regulation of the interplay between two specific cell types: bone-forming osteoblasts and bone-resorbing osteoclasts^1^. Imbalances in bone homeostasis cause severe bone diseases, including osteoporosis, the most prevalent chronic disease in aging adults. Bone homeostasis is affected by various factors, such as heart/vascular disease, estrogen deficiency, diabetes, and aging^2^. Aging is the main risk factor for age-related osteoporosis, which is characterized predominantly by decreased bone mineral density. The consequent bone structure fragility is a primary cause of osteoporotic fracture in elderly individuals^3,4^.

Bone-forming osteoblasts have a short lifespan and are constantly being replaced^5^. Osteoblast differentiation is governed by osteoblast-specific transcription factors, including runt-related transcription factor-2 (RUNX2)^6^ and osterix^7^. Differentiation consists of a sequence of events, including lineage commitment (osteoprogenitors), proliferation and matrix synthesis (preosteoblasts), and matrix maturation and mineralization (mature osteoblasts). Mature osteoblasts are responsible for the assembly of matrix proteins, including collagen type I (COL1A1), bone integrin-binding sialoprotein (IBSP), osteopontin (OPN) and osteocalcin (OCN)^8^, and are characterized by an increase in alkaline phosphatase (ALP) activity^9^. Age-related bone loss was shown to be caused by a deficit in bone formation^10^. Osteoblast dysfunction is the main cause of age-related bone loss in humans and results from two distinct types of mechanisms. Extrinsic mechanisms are mediated by changes in the bone microenvironment associated with hormones and growth factors, whereas intrinsic mechanisms are mediated by osteoblast senescence following reductions in the number, lifespan, and differentiation of osteoblasts^11^.

Age-related bone loss has also been associated with increases in the number of bone-resorbing osteoclasts^12^. Osteoclasts differentiate from hematopoietic progenitors of the monocyte-macrophage lineage in response to stimulation by macrophage colony-stimulating factor (M-CSF) and receptor activator of NF-κB ligand (RANKL)^13^. Osteoclasts develop into multinucleated giant cells through a sequence of steps that include proliferation, differentiation, and maturation^14,15^. Importantly, osteoclasts exhibit characteristics of senescence, such as the expression of several different cyclin-dependent kinase (CDK) inhibitors, INK4A (p16) and CDKN1A (p21), during osteoclast differentiation^16,17^. Osteocytes in the bones of aged mice are postmitotic and show elevated levels of p16^12,18^, whereas senescent osteoblast progenitors exhibit high levels of p21 but not p16^19^. Selective elimination of p16-expressing cells in mouse models increases bone mass^18^, and stromal cell cultures obtained from the bone marrow of old mice are better at promoting osteoclastogenesis than cells from young mice^20^, supporting the finding that senescent cells contribute to age-related bone loss.

Hypoxia-inducible factor (HIF), a heterodimeric transcription factor consisting of an oxygen-regulated α-subunit and a constitutively expressed β-subunit, plays important roles in bone formation by regulating the differentiation and activation of osteoblasts and osteoclasts^21^. Under normal oxygen tension, the α-subunit of HIF is hydroxylated by oxygen-sensitive prolyl hydroxylase, which is recognized by the von Hippel-Lindau protein, a component of the E3 ubiquitin ligase complex, and targeted for degradation^22^. Unlike HIF-1α, which is structurally very similar to HIF-2α but has many different functions^23^, HIF-2α can accumulate under normoxic conditions in the context of pathologies such as osteoporosis^24^ and arthritis^25–27^ and can also regulate bone remodeling^24^. HIF-2α depletion downregulates RANKL expression in fibroblast-like synoviocytes from the rheumatoid arthritis synovium^25^. In addition, HIF-2α regulates osteoblast and osteoclast differentiation via the expression of essential transcription factors involved in the differentiation of these cells and influences the interplay between these cell types^24^, suggesting that HIF-2α plays a catabolic role in bone remodeling. In this study, we tried to examine whether age-related upregulation of HIF-2α expression in osteoblasts and osteoclasts contributes to the differentiation of these cells, senescence progression and age-related bone loss.

## Materials and methods

### Mice

Male C57BL/6 J mice aged 1.5, 4, 12, and 20 months were purchased from Damool Science Animal Laboratory (Daejeon, Korea). Heterozygous *Hif-2α* KO (*Hif-2α*^+/−^) mice (#003266) and *Hif-2α*^fl/fl^ mice (#008407) were obtained from Jackson Laboratory (Sacramento, CA, USA). *Ctsk-*Cre mice were obtained from the Rodent Model Resource Center (RMRC13132, Taipei, Taiwan), and *Col1a1-*Cre mice were kindly provided by Dr. Je-Yong Choi (Kyungpook National University, Daegu, Korea). To generate osteoblast- and osteoclast-specific *Hif-2α-*KO mice, *Hif-2α*^fl/fl^ mice were backcrossed with *Col1a1-*Cre and *Ctsk-*Cre mice, respectively. Specific deletion of *Hif-2α* in targeted tissues in those mice was demonstrated in a previous study^24^. All animal experiments were approved by the Institutional Animal Care and Use Committee (IACUC) of Chonnam National University (Gwangju, Korea).

### Micro (μ)CT analysis and bone histomorphometry

Mouse femurs were fixed in 10% neutral buffered formalin and examined using μCT as previously described^24^. Trabecular morphometry was analyzed by measuring bone mineral density (BMD), bone volume/tissue volume (BV/TV), trabecular thickness (Tb.Th), trabecular separation (Tb.Sp), trabecular number (Tb.N), and cortical thickness (Ct.th). Three-dimensional surface rendering images were generated using Mimics 14.0 imaging software (Materialise, Plymouth, MI, USA). Histomorphometric parameters were analyzed by OsteoMeasure software (Osteometrics, Inc., Decatur, GA, USA). Paraffin sections were stained with hematoxylin and eosin (H&E) and BV/TV, the number of osteoblasts/bone perimeter (N.Ob/B.Pm), and the osteoblast surface/bone surface (Ob.S/BS) were analyzed. To investigate osteoclast parameters, the sections were stained with tartrate-resistant acid phosphate (TRAP) and the number of osteoclasts/bone perimeter (N.Oc/B.Pm) and the osteoclast surface/bone surface (Oc.S/BS) were measured.

### Osteoblast differentiation of primary cultured preosteoblasts

For primary culture of calvarial preosteoblasts, calvarial bones from 3-day-old pups were enzymatically digested twice with DMEM comprising 0.1% type II collagenase (Sigma–Aldrich, St. Louis, MO, USA) as described previously^24^. To induce cellular senescence in the proliferation stage, osteoblasts stimulated with doxorubicin on differentiation day 2 were treated with a potent HIF-2α inhibitor, TC-S 7009 (R&D Systems, Minneapolis, MN, USA) on differentiation day 4, and cells were harvested after 3 days. Cellular senescence in the matrix stage was induced by doxorubicin on differentiation day 9, and TC-S 7009 was administered on differentiation day 11 for 3 days. Then, the cells were harvested on day 14 of differentiation. A schematic diagram showing the time point at which each chemical compound was administered is provided in Supplementary Fig. 5. To overexpress *Hif-2α* in vitro, primary calvarial preosteoblasts were infected with adenovirus expressing *Hif-2α* at the indicated multiplicity of infection (MOI) on differentiation day 2. Doxorubicin is a commonly used chemotherapy drug that can induce DNA cross-linking and lead to cell apoptosis, autophagy, and/or senescence^28^. The concentration of doxorubicin used in this experiment did not cause cell apoptosis.

### Osteoclast differentiation of bone marrow macrophages (BMMs)

Mouse bone marrow cells were isolated and harvested as previously described^24^. Adherent BMMs (4 × 10^4^ cells per well in 48-well plates) were maintained in complete α-MEM containing 30 ng/ml M-CSF (PeproTech) for 24 h. Then, the medium was replaced with medium comprising 100 ng/ml RANKL (PeproTech), and the cells culture for 5 days. To ectopically overexpress HIF-2α, adenovirus encoding *Hif-2α* was applied at the indicated MOI on day 1. TRAP (Sigma–Aldrich) staining was performed on day 5 according to the manufacturer’s instructions.

### ALP and ARS staining

For ALP staining, cells were fixed with 4% paraformaldehyde for 2 min, washed with deionized water and stained with 5-bromo-4-chloro-3-indolyl phosphate (BCIP®)/nitro blue tetrazolium (NBT) liquid substrate solution (Sigma–Aldrich) for 15 min in a dark room. For mineralization assays, the cells were fixed with 10% formalin for 15 min and stained with 2% Alizarin red S (ARS, Sigma–Aldrich) solution for 45 min at room temperature.

### Reverse transcription-polymerase chain reaction (RT-PCR) and quantitative real-time (qRT)-PCR

Mouse bone tissues were immediately frozen in liquid nitrogen and stored at −70 °C until use. Total RNA was isolated from osteoblasts, osteoclasts, and femoral bone tissues using TRI reagent (Molecular Research, Cincinnati, OH, USA) and reverse-transcribed with TOPscript RT DryMIX (Enzynomics, Daejeon, Korea). cDNA was subjected to PCR using AmpOne^TM^ Tap DNA Polymerase Mix (GeneAll, Seoul, Korea) and appropriate primers. qPCR was performed using a StepOnePlus Real-Time PCR system (Thermo Fisher Scientific, Waltham, MA, USA) and SYBR Premix Ex Taq (TaKaRa Bio, Kyoto, Japan). The individual transcript levels of each individual target were normalized to those of *Gapdh* and are expressed as the fold change relative to the levels in the indicated controls. The primer sequences and sizes of the amplicons are listed in Supplementary Table 1.

### Western blotting

Cells were lysed in lysis buffer (50 mM Tris-HCl, pH 8.0, 150 mM NaCl, 5 mM NaF, 1% NP-40, 0.2% SDS, and 0.5% deoxycholate) supplemented with protease and phosphatase inhibitor cocktails (Roche, Basel, Switzerland). Proteins were separated by SDS-PAGE, and the blots were incubated with rabbit anti-HIF-2α (NB100-122; Novus Biologicals, CO, USA) at 4 °C overnight. After washing, the membranes were incubated with HRP-conjugated anti-rabbit IgG, and images were obtained using ImageSaver6 software via the EZ-capture MG system (ATTO, Tokyo, Japan). The protein bands were quantified using a CS analyzer 4 (ATTO), and protein levels were normalized relative to the level of β-actin (Sigma–Aldrich) in the same sample.

### Immunohistochemistry and immunofluorescence microscopy

Sagittal Section (5-μm thick) were placed on slides for immunohistochemical staining. The sections were incubated in 3% H_2_O_2_ for 10 min to block endogenous peroxidase activity and incubated in 0.1% trypsin for 30 min at 37 °C for antigen retrieval. After blocking with 1% BSA for 30 min, the slides were reacted with rabbit anti-HIF-2α (Novus Biologicals), mouse anti-p16 (MA5-17142, Invitrogen, Carlsbad, CA, USA), and mouse anti-p21 (AHZ0422, Invitrogen) antibodies, stained using EnVision+System-HRP (Dako, Denmark) and AEC+ substrate and counterstaining with hematoxylin (Dako). The primary antibodies used for double immunofluorescence staining of mouse tissues included mouse anti-HIF-2α (sc13596; Santa Cruz Biotech., Dallas, TX, USA), rabbit anti-OCN (AB10911; Millipore, Burlington, MA, USA), and rabbit anti-cathepsin K (ab19027; Abcam, Cambridge, MA, USA) antibodies. Proteins were visualized using Alexa 488- or Alexa 594-conjugated secondary antibodies (Thermo Fisher Scientific). Nuclei were visualized with DAPI.

### Enzyme-linked immunosorbent assay (ELISA)

Serum OCN and CTX-1 levels were measured using a mouse Gla-OCN High Sensitive EIA Kit (MK127; TaKaRa Bio) and CTX-1 EIA Kit (NBP2-69074; Novus Biologicals). All procedures were performed according to the manufacturers’ instructions.

### Chromatin immunoprecipitation (ChIP) assay

ChIP assays were performed using a Magna ChIP Kit (Millipore) as previously described^24^. Briefly, cells were fixed with 1% formaldehyde to crosslink the DNA and protein complexes. After the lysates were sonicated, soluble chromatin complexes were incubated with rabbit anti-HIF-2α antibody or anti-IgG at 4 °C overnight. The precipitated DNA segment of the *p16* or *p21* promoter was amplified by RT-PCR. The sequences of the primers used for the ChIP assay are listed in Supplementary Table 1.

### Statistical analysis

All experiments were repeated at least three times. The results are reported as the mean ± SD. μCT and histomorphometric parameters are presented in bar graphs with scatter plots. All statistical analyses were performed using GraphPad Prism version 7 (GraphPad, Inc., San Diego, CA). All quantitative data were first tested for normal distribution using the Shapiro-Wilk test. Then, the means of quantitative variables were compared between two groups by two-tailed Student’s *t*-test or between three or more independent groups by analysis of variance (ANOVA) followed by Bonferroni’s post-hoc test (multicomparison), as appropriate. *n* is the number of independent experiments or mice. The results were considered statistically significant when the *p-*value was less than 0.05.

## Results

### HIF-2α expression is upregulated in skeletal bones with aging

**Fig. 1:**
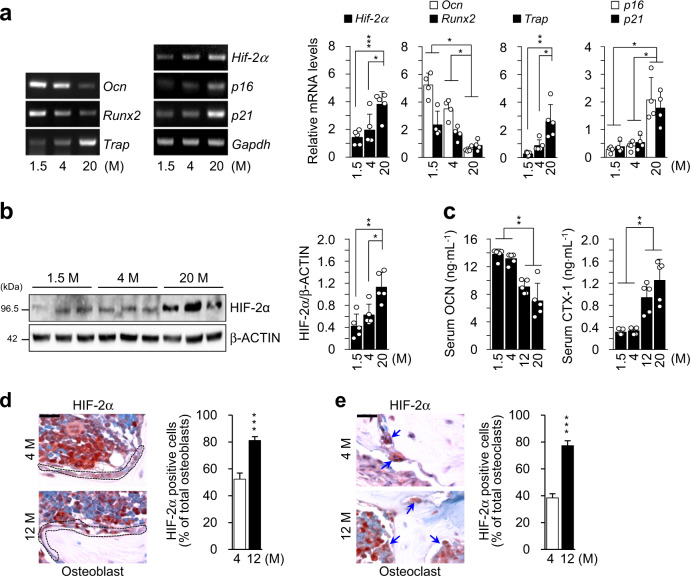
HIF-2α expression is upregulated in skeletal bones with aging. **a** Detection of the indicated mRNAs in the long bones of 1.5-, 4-, and 20-month-old mice. The levels of mRNAs encoded by *Hif-2α*, osteoblast marker genes (*Ocn* and *Runx2*), an osteoclast marker gene (*Trap*) and senescence marker genes (*p16* and *p21*) were determined by RT-PCR and quantified by qRT-PCR (*n* ≥ 4). **b** The protein levels of HIF-2α extracted from mice of the indicated age determined by western blot analysis (left panel) and quantified by a CS analyzer 4 (right panel) (*n* = 5). **c** ELISA-based measurements of the serum concentrations of OCN (*n* = 5) and CTX-1 (*n* = 5) in 1.5-, 4-, 12- and 20-month-old mice. **d,** **e** Immunostaining of osteoblasts (**d**) and osteoclasts (**e**) from the bone tissues of young (4-month-old) and old (12-month-old) mice for HIF-2α. The dotted lines indicate osteoblasts, and the arrowheads indicate osteoclasts. Scale bar: 25 μm. Quantification of HIF-2α-positive cells is shown in the right panel (*n* = 8). M = month. The values are presented as the means ± SDs (*P < 0.05, **P < 0.01, and ***P < 0.005).

*Hif-2α* expression was markedly higher in the aged bones of 20-month-old male mice than in the bones of younger mice and exhibited the same pattern of expression as the aging-related markers *p16* and *p21* (Fig. 1a). Because bone tissue is a complex system that consists of diverse cell types, including osteoblasts, osteocytes and osteoclasts along with various supporting cells, including hematopoietic cells, the upregulated *Hif-2α* expression shown in Fig. 1a, b might be associated with various cell types. Here, we focused on a representative bone remodeling process maintained by the resorption of osteoclasts and performed by osteoblasts. The expression of the osteoblast markers *Ocn* and *Runx2* was lower in the bones of aged mice than in those of younger mice, while the expression of *Trap*, a marker of osteoclasts, was dramatically increased in aged bones (Fig. 1a). Consistent with the level of *Hif-2α* mRNA, HIF-2α protein levels were significantly higher in the bones aged mice than in those of younger mice (Fig. 1b). To evaluate bone turnover in aged mice, the levels of biochemical markers in the serum were compared between mice at different ages. Serum OCN levels were lower but the level of the bone resorption-specific biomarker serum CTX-1 was higher in 12- and 20-month-old male mice than in 1.5- and 4-month-old male mice (Fig. 1c). To confirm the in vivo involvement of HIF-2α expression and bone turnover in aged bone cells, the numbers of HIF-2α-expressing osteoblasts (Fig. 1d) and osteoclasts (Fig. 1e) were quantified histochemically in young (4-month-old) and aged (12-month-old) mice. The number of HIF-2α-positive osteoblasts were higher in the bone tissue of aged mice than in that of younger mice (Fig. 1d). The number of HIF-2α-expressing osteoclasts was increased in aged mice (Fig. 1e). These findings indicated that HIF-2α expression was upregulated in both osteoblasts and osteoclasts upon aging.

### Age-induced osteoporotic bone loss is reversed in heterozygous *Hif-2α* knockout (KO) mice

**Fig. 2:**
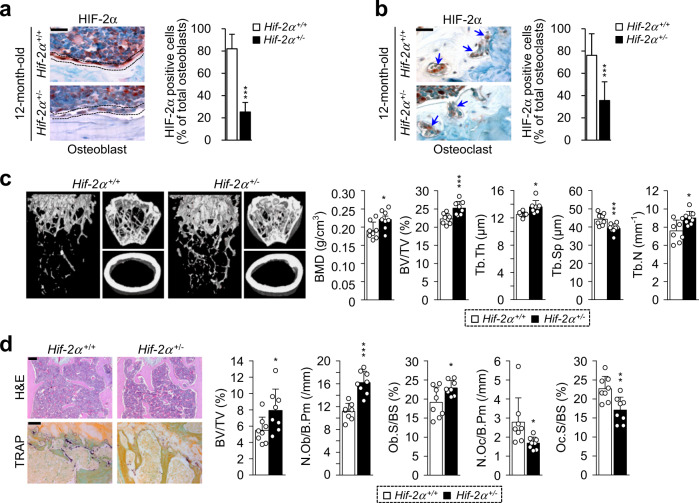
Heterozygous *Hif-2α* KO mice show reversal of aging-induced bone loss. **a**, **b** Representative images of osteoblasts (**a**) and osteoclasts (**b**) from the bone tissues of WT (*Hif-2α*^+/+^) and *Hif-2α*^+/−^ mice stained with an anti-HIF-2α antibody. The dotted lines indicate osteoblasts, and the arrowheads indicate osteoclasts. Scale bar: 25 μm. Quantification of HIF-2α-positive osteoblasts and osteoclasts is shown in the right panels (*n* = 8). **c**, **d** Analysis of femoral trabecular bones from 12-month-old *Hif-2α*^+/−^ and WT mice. Representative images of µCT reconstructions of femoral trabecular and cortical bones (**c**) and H&E and TRAP staining of trabecular bones (Scale bar: 100 μm; **d**). Bone mineral density (BMD), bone volume per tissue volume (BV/TV), trabecular bone thickness (Tb.Th), trabecular separation (Tb.Sp), and trabecular number (Tb.N) determined from µCT measurements (*n* = 8; **c**). BV/TV, the number of osteoblastic cells per bone perimeter (N.Ob/B.Pm), the osteoblast surface normalized to the bone surface (Ob.S/BS), the number of osteoclastic cells per bone perimeter (N.Oc/B.Pm), and the osteoclast surface normalized to the bone surface (Oc.S/BS) were assessed by bone histomorphometric analyses of the metaphyseal regions of femurs (*n* = 8; **d**). The values are presented as the means ± SDs (*P < 0.05, **P < 0.01, and ***P < 0.005).

The association between upregulation of HIF-2α expression and aged-related bone loss was investigated by examining the bone microarchitecture of 12-month-old male heterozygous *Hif-2α* KO (*Hif-2α*^+/−^) and wild-type (*Hif-2α*^+/+^) mice using X-ray microcomputed tomography (μCT). Immunohistochemical staining with anti-HIF-2α and anti-OCN antibodies confirmed that *Hif-2α* was depleted of osteoblasts in *Hif-2α* KO mice (Fig. 2a and Supplementary Fig. 1a). Since OCN is expressed only in osteoblasts, osteoblasts can be distinguished from bone lining cells, which do not express OCN^29^. Knockdown of HIF-2α expression in the osteoclasts of *Hif-2α* KO mice was also verified by immunohistochemistry (Fig. 2b). Consistent with our previous findings^24^, quantitative results of bone parameters indicated that the bone mass and trabecular bone percentages were higher in 4-month-old *Hif-2α* KO mice than in age-matched WT mice (Supplementary Fig. 1b). Quantitative μCT analyses showed that trabecular bone volume was significantly decreased in aged mice (12 months old) compared to young mice (4 months old) (Supplementary Fig. 1b) and that cancellous trabeculae in the femoral bone were better preserved in *Hif-2α*^+/−^ mice than in WT littermates (Fig. 2c). Trabecular bone volume was higher in *Hif-2α*^+/−^ mice than in WT mice, as measured by BMD and BV/TV (Fig. 2c). Other bone parameters, including Tb.Th and Tb.N, were higher, whereas Tb.Sp was lower, in *Hif-2α*^+/−^ mice than in WT mice (Fig. 2c). No differences in cortical thickness were detected between *Hif-2α*^+/−^ mice and age-matched WT mice at any age (Supplementary Fig 1b). The cause of this discrepancy may be the fact that cortical bone is generally less metabolically active than trabecular bone^30,31^. Therefore, we focused on analyzing trabecular bone physiology. Bone histomorphometric analyses showed that the bone trabecular percentage was higher and that the number of osteoclasts was lower in heterozygous *Hif-2α* KO mice than in WT mice (Fig. 2d). BV/TV, N.Ob/B.Pm, and Ob.S/BS were increased in *Hif-2α*^*+/−*^ mice, whereas N.Oc/B.Pm and Oc.S/BS, which are parameters associated with bone resorption, were decreased in *Hif-2α*^*+/−*^ mice (Fig. 2d). These data indicated that HIF-2α deficiency retarded bone destruction in aged mice.

### Upregulation of HIF-2α expression is associated with osteoblast dysfunction upon aging

**Fig. 3:**
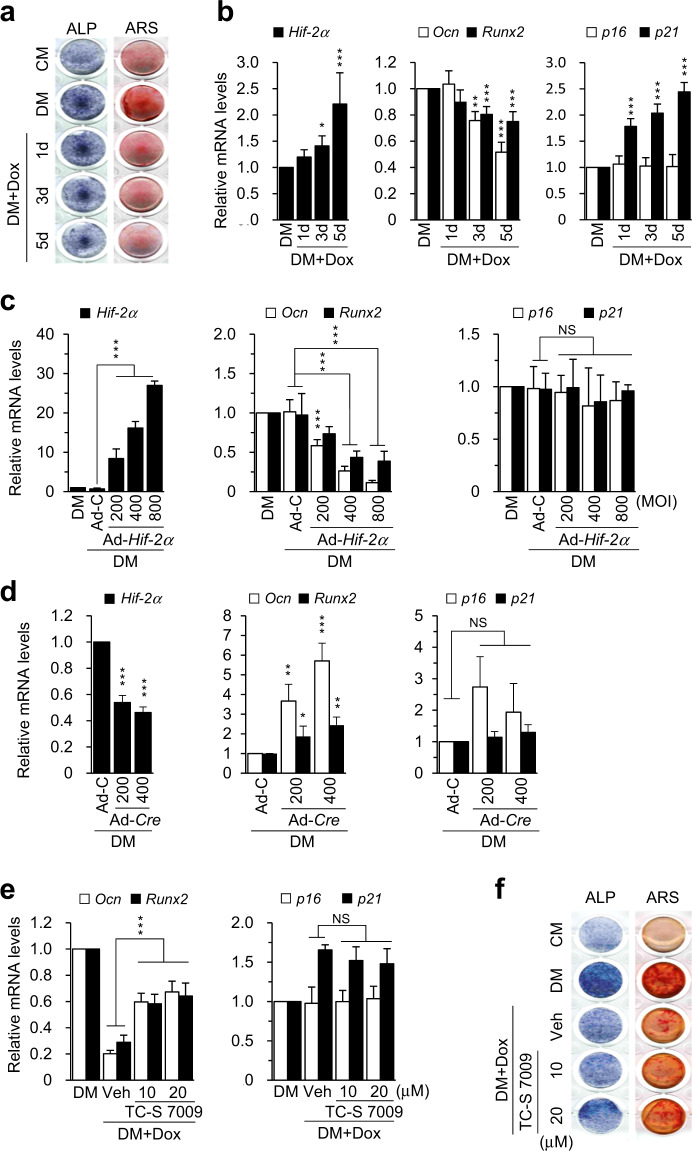
Upregulation of HIF-2α expression by aging inhibits osteoblast function. **a**, **b** Doxorubicin-induced inhibition of osteoblast differentiation. Primary calvarial preosteoblasts from newborn mice were cultured in osteogenic differentiation medium (DM) containing 50 μg/ml L-AA and 5 mM β-GP for 7 days. Before harvesting cells on day 14, 0.01 μg/ml doxorubicin was added to the differentiation medium for 1, 3, or 5 days. Representative images of alkaline phosphatase (ALP) and alizarin red S (ARS) staining in primary calvarial preosteoblasts cultured in control medium (CM) or differentiation medium in the absence or presence of 0.01 μg/ml doxorubicin are shown (**a**). The expression levels of *Hif-2α, Ocn, Runx2, p16*, and *p21* were analyzed by qRT-PCR (*n* = 5; **b**). **c** Quantification of mRNA levels by qRT-PCR. The transcript levels of the indicated genes were measured in primary calvarial preosteoblasts infected with Ad-C at an MOI of 800 or Ad*-Hif-2α* at the indicated MOI (*n* ≥ 4). **d** The mRNA levels of *Hif-2α, Ocn, Runx2, p16*, and *p21*. Osteoblasts isolated from *Hif-2α*^fl/fl^ mice were infected with Ad-C or Ad-*Cre* in the presence of differentiation medium (*n* ≥ 4). **e**, **f** Effect of HIF-2α inhibition on osteoblast matrix maturation. Doxorubicin-treated osteoblasts were treated with the potent HIF-2α inhibitor TC-S 7009 and incubated for 3 days. The levels of *Ocn*, *Runx2, p16*, and *p21* mRNAs were measured by qRT-PCR (*n* = 5; **e**). Assessment of osteoblast differentiation and mineral deposition by ALP and ARS staining (**f**). The values are presented as the means ± SDs (*P < 0.05, **P < 0.01, and ***P < 0.005; NS not significant).

The effect of increased HIF-2α expression in osteoblasts during age-related bone loss was investigated by assessing the effects of HIF-2α on osteoblast differentiation and senescence. OCN was found to be rarely expressed in HIF-2α-expressing osteoblasts from the bones of 12-month-old mice (Supplementary Fig. 2), suggesting that increased HIF-2α expression in aged bones might be associated with osteoblast senescence, resulting in an imbalance in bone homeostasis and age-mediated bone loss. To assess this hypothesis, doxorubicin-induced senescence during mouse osteoblast differentiation was analyzed. Two different experimental conditions were tested to determine the stage of osteoblast differentiation affected by doxorubicin-induced senescence. Generally, differentiation from preosteoblasts to mature osteoblasts is divided into two stages, proliferation, which occurs in the first 7 days, and matrix maturation, which occurs over the subsequent 7 days, depending on the duration of culture^32,33^. The protein expression of HIF-2α increased during osteoblast proliferation, peaked during matrix maturation and was observed until day 14 (data not shown). The addition of doxorubicin during both phases effectively blocked L-ascorbic acid (L-AA) and β-glycerophosphate (β-GP)-induced differentiation of preosteoblasts, as determined by measurement of ALP activity and ARS staining (Fig. 3a and Supplementary Fig. 3a). Induction of doxorubicin-mediated osteoblast senescence, as determined by increased *p21* expression but not *p16* expression, resulted in the upregulation of *Hif-2α* expression and the downregulation of expression of the osteoblastic marker genes *Ocn* and *Runx2* (Fig. 3b and Supplementary Fig. 3b, c). To evaluate whether overexpression of HIF-2α during osteoblast differentiation affects the progression of senescence, calvarial preosteoblasts were infected with adenovirus expressing *Hif-2α* (Ad-*Hif-2α*), which significantly inhibited the expression of *Ocn* and *Runx2* (Fig. 3c and Supplementary Fig. 3d). However, overexpression of *Hif-2α* did not affect the expression of the senescence marker genes *p16* and *p21*, indicating that *Hif-2α* expression did not induce senescence (Fig. 3c and Supplementary Fig. 3d). This result was additionally confirmed by osteoblast-specific *Hif-2α* knockout by Ad-*Cre* infection in osteoblasts obtained from *Hif-2α*^fl/fl^ mice. Consistent with the alteration of the expression of genes, including *Hif-2α*, *Ocn*, and *Runx2*, the expression of the senescence marker genes *p16* and *p21* was not affected (Fig. 3d). TC-S 7009 is a potent inhibitor of HIF-2α activity, and its inhibitory effect was confirmed by its ability to suppress IL-1β- and Ad-*Hif-2α*-induced HIF-2α activation in a reporter gene assay using a 4×HRE-containing luciferase vector construct (Supplementary Fig. 4). TC-S 7009-mediated inhibition of HIF-2α activity during the osteoblast proliferation phase failed to recover osteoblast differentiation inhibited by senescence (Supplementary Fig. 5a, b). However, HIF-2α inhibition during matrix maturation resulted in significant reversal of the doxorubicin-mediated reduction in *Ocn* and *Runx2* expression without altering *p16* expression (Fig. 3e and Supplementary Fig. 5c). Moreover, doxorubicin-mediated inhibition of osteoblast differentiation was reversed by TC-S 7009-induced inhibition of HIF-2α, as determined by ALP and ARS staining (Fig. 3f and Supplementary Fig. 5d). These findings indicated that increased HIF-2α expression was an effect of aging rather than a cause of osteoblast senescence in aged bones. However, inhibition of HIF-2α was able to restore age-related impaired osteoblast matrix maturation by increasing the expression of genes such as *Ocn* and *Runx2*.

### Osteoblast-specific depletion of HIF-2α increases bone mass in aged mice

**Fig. 4:**
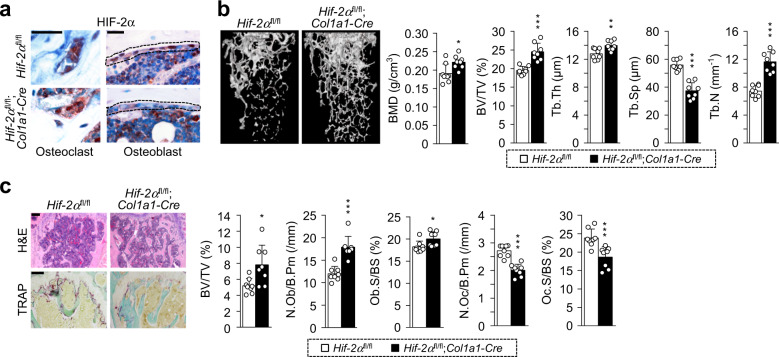
Osteoblast-specific depletion of HIF-2α reverses aging-related bone loss. **a** Representative images of HIF-2α in osteoclasts (left panel) and osteoblasts (right panel) from *Hif-2α*^fl/fl^ and *Hif-2α*;^fl/fl^*Col1a1*-*Cre* mice. The dotted lines indicate osteoblasts. Scale bar: 25 μm. **b**, **c** Analyses of femoral trabecular bones from 12-month-old *Hif-2α*^fl/fl^ and *Hif-2α*;^fl/fl^*Col1a1*-*Cre* mice. Representative images of µCT reconstructions of trabecular bones (**b**) and H&E and TRAP staining (**c**) are presented. BMD, BV/TV, Tb.Th, Tb.Sp, and Tb.N were determined from µCT measurements (*n* = 8; **b**). Determination of BV/TV, N.Ob/B.Pm, Ob.S/BS, N.Oc/B.Pm and Oc.S/BS from bone histomorphometric analysis of the metaphyseal regions of femurs (*n* = 8; **c**). Scale bar: 100 μm. The values are presented as the means ± SDs (*P < 0.05; **P < 0.01, and ***P < 0.005).

To further investigate the association between HIF-2α expression upregulation in osteoblasts and age-mediated osteoporotic bone loss, male osteoblast-specific *Hif-2α* conditional KO (*Hif-2α*;^fl/fl^*Col1a1*-*Cre*) mice were analyzed. HIF-2α was specifically depleted in the osteoblasts of these mice (Fig. 4a). μCT images showed that aging-induced trabecular bone loss in 12-month-old *Hif-2α*;^fl/fl^*Col1a1*-*Cre* mice (Supplementary Fig. 6) was much lower than that in age-matched *Hif-2α*^fl/fl^ mice (Fig. 4b). Quantitative analyses showed that BMD, BV/TV, Tb.Th and Tb.N were higher and that Tb.Sp was lower in osteoblast-specific *Hif-2α*-deficient mice than in *Hif-2α*^fl/fl^ mice (Fig. 4b). Consistent with these μCT images, H&E staining and bone histomorphometric parameters, such as BV/TV, N.Ob/B.Pm and Ob.S/BS also showed that the trabecular percentage was increased in *Hif-2α*;^fl/fl^*Col1a1*-*Cre* mice (Fig. 4c). The results of TRAP staining and measurement of quantitative parameters associated with bone resorption, such as N.Oc/B.Pm and Oc.S/BS, revealed that bone resorption was decreased in aged *Hif-2α*;^fl/fl^*Col1a1*-*Cre* mice (Fig. 4c). These findings indicated that osteoblast-specific depletion of HIF-2α delayed age-induced osteoporotic bone loss.

### Aging-associated upregulation of HIF-2α expression accelerates osteoclast function and senescence

**Fig. 5:**
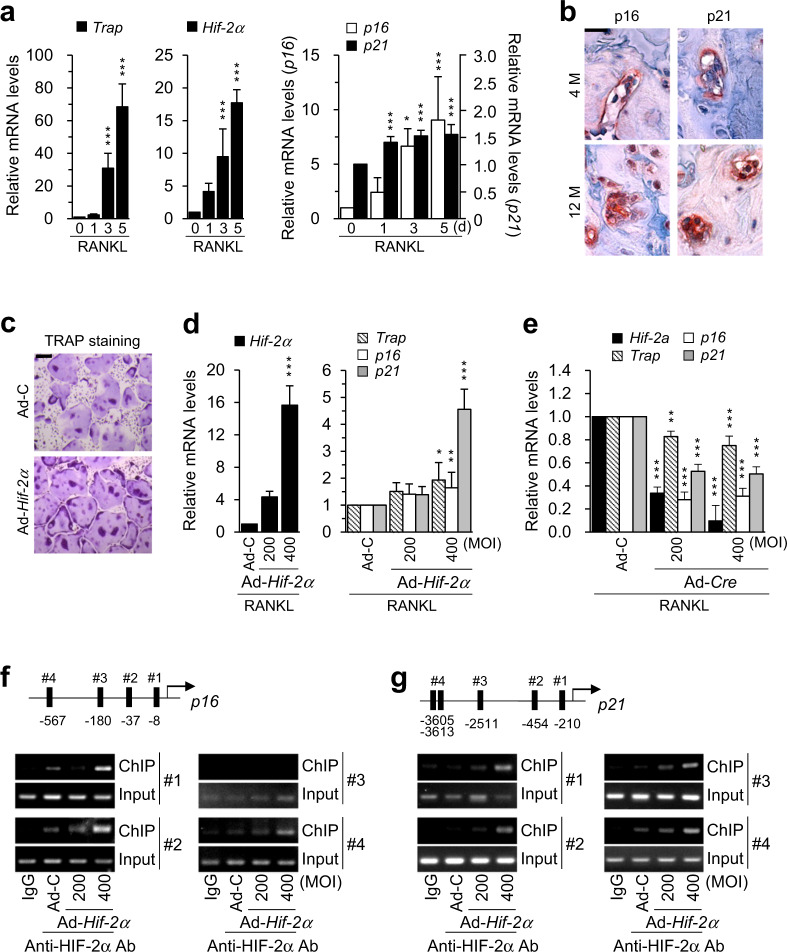
Upregulation of HIF-2α expression by aging stimulates osteoclastogenesis and osteoclast senescence. **a** The mRNA levels of osteoclast marker genes (*Trap*), *Hif-2*α, and senescence markers (*p16*, *p21*) during M-CSF/RANKL-induced osteoclastogenesis of bone marrow macrophages (BMMs) (*n* ≥ 3). **b** Representative images of p16 and p21 immunostaining in bone osteoclasts from young (4-month-old) and old (12-month-old) mice. Scale bar: 25 μm. M month. **c,** **d** TRAP staining (**c**) and qRT-PCR analysis of *Hif-2α*, *Trap*, *p16*, and *p21* expression (*n* ≥ 4; **d**) in *Hif-2*α-overexpressing osteoclasts. BMMs were infected with Ad-C or Ad-*Hif-2α* and cultured with M-CSF and RANKL for 5 days. Scale bar: 100 μm. **e** The mRNA levels of *Hif-2α*, *Trap, p16*, and *p21*. Osteoclasts isolated from *Hif-2α*^fl/fl^ mice were infected with Ad-C or Ad-*Cre* during osteoclastogenesis (*n* ≥ 4). **f,** **g** Binding of HIF-2α to the promoter regions of *p16* (**f**) and *p21* (**g**). Ad-*Hif-2α*-infected osteoclasts were subjected to ChIP with an anti-HIF-2α antibody and a primer pair designed to span the putative HIF-2α binding regions [5’-(A/G)CGTG-3’] within the promoters of *p16* (*n* = 3; **f**) and *p21* (*n* = 3; **g**). The values are presented as the means ± SDs (*P < 0.05, **P < 0.01, and ***P < 0.005).

Because age-mediated disruption of bone homeostasis is also caused by increased osteoclast activity^34^, the effect of aging on HIF-2α expression in osteoclasts was assessed. HIF-2α was strongly expressed in CTSK-positive osteoclasts in the bones of 12-month-old mice (Supplementary Fig. 7a). *Hif-2α* expression was increased on days 3–5 of in vitro RANKL-induced osteoclast differentiation of BMMs, consistent with the increases in *p16* and *p21* expression (Fig. 5a and Supplementary Fig. 7b). Immunohistochemistry also showed that the expression of *p16* and *p21* was higher in osteoclasts from the bones of 12-month-old mice than those from the bones of younger mice (Fig. 5b). Assessment of the direction contribution of HIF-2α to the aging of osteoclasts showed that osteoclastogenesis was significantly enhanced by transduction with Ad-*Hif-2α*, as evidenced by an increase in the number of TRAP-positive multinucleated cells (Fig. 5c). Interestingly, *p16* and *p21* expression was concomitantly enhanced by the overexpression of *Hif-2α* during RANKL-induced osteoclast differentiation of BMMs (Fig. 5d and Supplementary Fig. 7c). Conversely, HIF-2α knockdown in BMMs obtained from *Hif-2α*^fl/fl^ mice by Ad-*Cre* infection led to the downregulation of *p16* and *p21* expression (Fig. 5e). To determine whether *p16* and *p21* are specific targets of HIF-2α, putative HIF-2α binding sites [5’-(A/G)CGTG-3’] were identified within the promoter region of these two genes (Fig. 5f, g). ChIP assays using four primer pairs designed to span these putative binding sites revealed that HIF-2α directly regulated the expression of both *p16* and *p21* (Fig. 5f, g). These findings indicated that increased HIF-2α expression in aged osteoclasts promoted the expression of *p16* and *p21* and mediated the senescence-related enhancement of the bone resorption activity of osteoclasts.

### Osteoclast-specific depletion of HIF-2α inhibits bone loss in aged mice

**Fig. 6:**
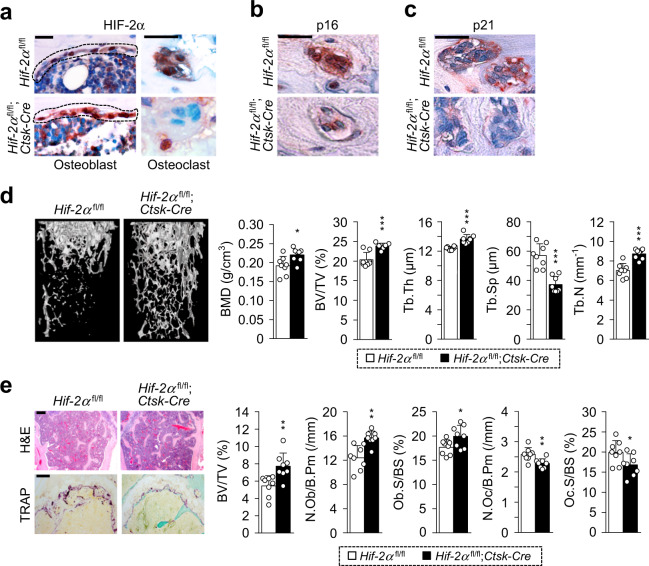
Osteoclast-specific depletion of HIF-2α increases bone mass in aged mice. **a**–**c** Immunostaining of HIF-2α, p16, and p21 in *Hif-2α*^fl/fl^ and *Hif-2α*;^fl/fl^*Ctsk*-*Cre* mice. Osteoclast-specific depletion of HIF-2α in 12-month-old *Hif-2α*^fl/fl^ and *Hif-2α*;^fl/fl^*Ctsk*-*Cre* mice was assessed by immunohistochemistry with anti-HIF-2α antibody. The dotted lines indicate osteoblasts (Scale bar: 25 μm; **a**). Immunostaining of p16 (**b**) and p21 (**c**) in osteoclasts from *Hif-2α*^fl/fl^ and *Hif-2α*;^fl/fl^*Ctsk*-*Cre* mice was examined by immunofluorescence microscopy. Scale bar: 25 μm. **d** Quantitative µCT analysis of trabecular bones. BMD, BV/TV, Tb.Th, Tb.Sp, and Tb.N in trabecular bones from 12-month-old *Hif-2α*^fl/fl^ and *Hif-2α*;^fl/fl^*Ctsk-Cre* mice (*n* = 8). **e** Representative images of H&E and TRAP staining in 12-month-old *Hif-2α*^fl/fl^ and *Hif-2α*;^fl/fl^*Ctsk-Cre* mice (*n* = 8; Scale bar, 100 μm). BV/TV, N.Ob/B.Pm, Ob.S/BS, N.Oc/B.Pm, and Oc.S/BS were determined by bone histomorphometric analyses of the metaphyseal regions of femurs. The values are presented as the means ± SDs (*P < 0.05, **P < 0.01, ***P < 0.005).

The association between HIF-2α expression upregulation in osteoclasts and age-mediated osteoporotic bone loss was further analyzed in osteoclast-specific male *Hif-2α* conditional KO (*Hif-2α*;^fl/fl^*Ctsk*-*Cre*) mice. Depletion of HIF-2α in osteoclasts but not osteoblasts was verified by immunohistochemical staining (Fig. 6a). Immunohistochemistry also showed that p16 (Fig. 6b) and p21 (Fig. 6c) expression was downregulated in *Hif-2α*-deficient osteoclasts. Quantitative analyses revealed that 12-month-old mice showed much lower trabecular bone mass than 4-month-old mice (Supplementary Fig. 8). Osteoclast-specific depletion of *Hif-2α* prevented this reduction in trabecular bone loss, as determined by μCT imaging and analyses of quantitative parameters, such as BMD, BV/TV, Tb.Th, Tb.Sp, and Tb.N (Fig. 6d). Bone histomorphometric analyses by H&E and TRAP staining showed that N.Oc/B.Pm and Oc.S/BS was significantly decreased in *Hif-2α*;^fl/fl^*Ctsk*-*Cre* mice, whereas N.Ob/B.Pm and Ob.S/BS were increased (Fig. 6e). These in vivo data showed that specific depletion of HIF-2α in osteoclasts delayed age-induced osteoporotic bone loss.

## Discussion

As aging progresses, bone remodeling, which is normally tightly regulated, may be dysregulated due to imbalances between osteoblast-mediated bone formation and osteoclast-mediated bone resorption. This imbalance can result in an increased risk of fracture, impaired bone healing, and the chronic age-related bone metabolism disease osteoporosis. This study showed that senescence-induced HIF-2α expression upregulation in both osteoblasts and osteoclasts acts as a novel intrinsic mediator of age-related bone loss.

The senescence-related genes *p16* and *p21* play important roles in the initiation of senescence by leading to cell cycle arrest^11^. Doxorubicin-induced senescence during osteoblastic differentiation of osteoblast progenitor cells resulted in increased expression of *p21* but not *p16*. In the absence of doxorubicin-mediated induction of senescence, however, both genes were highly expressed in mature osteoclasts. Upregulated HIF-2α expression in *p21*-expressing senescent osteoblasts inhibited osteoblast differentiation and function, as determined by decreases in *Ocn* and *Runx2* expression. *Twist2*, a gene directly targeted by HIF-2α, has been shown to be expressed during early stages of osteogenic differentiation and to inhibit the expression of RUNX2, a key transcription factor for osteoblast differentiation, resulting in decreased expression of OCN, which has been implicated in bone mineralization^24^. TWIST is a negative regulator of RUNX2 expression and function that downregulates OCN expression^35^, whereas doxorubicin enhances TWIST expression in mouse osteoblastic cells^36^. The current study hypothesized that upregulated HIF-2α expression in osteoblasts in aged bones suppresses RUNX2 expression by inducing TWIST2 expression upregulation, resulting in the sequential downregulation of OCN expression. Senescent cells can cause tissue dysfunction through the development of senescence-associated secretory phenotypes, which are characterized by the production of extracellular matrix-degrading proteins, proinflammatory cytokines and chemokines^37,38^. These aging-associated changes in the bone microenvironment may induce increased HIF-2α expression in osteoblasts. Indeed, unlike that of HIF-1α, the expression of HIF-2α can be enhanced by proinflammatory cytokines, including IL-1β and TNFα, in the absence of hypoxia^26^. In general, the osteoblast differentiation process can be divided into three phases: proliferation, matrix maturation, and mineralization^39^. We previously reported that HIF-2α is highly expressed until the matrix maturation stage and that HIF-2α expression declines during later stages of osteogenic differentiation^24^ .The present study showed that doxorubicin inhibited osteoblast differentiation during both the proliferation and matrix maturation stages. In contrast, HIF-2α inhibited the maturation process but not the proliferation, thereby controlling osteoblast differentiation. These unexpected discrepancies may be related to the altered expression of differentiation-associated genes, differences in accessibility of the promoters of HIF-2α target genes during the complicated differentiation process, or the sensitivity of the HIF-2α inhibitor employed. Further studies are needed to assess the catabolic effect of HIF-2α on age-related osteoblast dysfunction in mediating age-related reductions in bone mass.

This study also revealed that the expression levels of HIF-2α, p16, and p21 were increased in osteoclasts and that their expression levels were higher in the bones of aged mice than in those of younger mice. Upregulated HIF-2α expression in osteoclasts promoted the transcription of *p16* and *p21* by binding directly to hypoxia-responsive elements (HREs; -(A/G)CGTG-) in the promoters of these genes. Thus, low p16 and p21 expression was detected in *Hif-2α*;^fl/fl^*Ctsk*-*Cre* mice. Upregulation of HIF-2α expression in senescent osteoclasts predominantly enhances bone-resorbing activity, as evidenced by the fact that inducible elimination of *p16*-expressing senescent cells in *INK-ATTAC* transgenic mice is associated with decreases in the number of osteoclasts and bone resorption^40^. In addition, senescent cell-condition medium enhances osteoclastogenesis and bone-resorbing activity and impairs osteoblast mineralization^40^. In our current study, the degree of cell senescence was assessed by measuring the expression levels of *p16* and *p21* because it is well established that these genes are the most well-known effectors of senescence and that their expression levels increase with age in several rodent and human cell lines^4,18,41^. In addition, Saeed et al. demonstrated that *p16* and *p21* levels are significantly increased in bone cells from telomerase knockout mice, which are premature aging model mice^42^. Although these genes act as key effectors of cellular senescence, since the phenomena caused by aging are diverse, additional investigations of other factors are necessary.

Bone is affected by weight load. The spine and legs, which support the human body, show marked changes in shape, such as bending of the bones, with aging. However, in mice, since the body is supported by four limbs, the morphological and physiological changes associated with age-related bone loss in the femur might be more serious than those in vertebrae. In addition, in murine models, weight-bearing bone is highly sensitive to running, which is a weight-bearing exercise^43,44^. Furthermore, some studies that observed aged bone showed no differences in age-related bone loss between femurs and vertebrae^45,46^. Therefore, in this study, we measured bone parameters in the femurs, not the spines, of mice.

HIF-2α deficiency in aged male mice markedly reversed age-related bone loss, which is consistent with our previous results showing that HIF-2α depletion in mice increases bone mass by promoting osteoblast differentiation and function and by inhibiting osteoclast differentiation and maturation^24^. Interestingly, both *Hif-2α*;^fl/fl^*Col1a1*-*Cre* and *Hif-2α*;^fl/fl^*Ctsk*-*Cre* male mice showed relatively better preservation of trabeculae than age-matched *Hif-2α*^fl/fl^ mice due to changes in both osteoblasts and osteoclasts. Osteoblast-mediated bone formation was higher and osteoclast-mediated bone resorption was lower in the bones of 12-month-old *Hif-2α*;^fl/fl^*Col1a1*-*Cre* and *Hif-2α*;^fl/fl^*Ctsk*-*Cre* conditional KO mice than in the bones of control mice, which may have been due to interactions between osteoblasts and osteoclasts. Osteoblasts influence osteoclast formation and differentiation through OPG/RANKL/RANK and LGR4/RANKL/RANK signaling^47,48^. RANKL expressed by osteoblasts can promote osteoclastogenesis by stimulating the RANK receptor on osteoclasts, a process that is regulated by the ratio of RANKL to OPG, a natural antagonist of RANK^49^. We previously reported that *Rankl* is a direct target gene of HIF-2α and that RANKL expression induced by HIF-2α overexpression promotes osteoblast-mediated osteoclast differentiation^24^. We hypothesized that osteoblast-specific HIF-2α depletion might reduce RANKL secretion by osteoblasts, reducing the RANKL/OPG ratio and the number of osteoclasts. Moreover, osteoclasts can affect osteoblast-mediated bone formation via Atp6v0d2^50^, complement component 3a^47^, semaphorin 4D^51^, or exosomal miR-214-3p^52^. Because TRAF6, which acts as a crucial adaptor protein of RANK signaling in osteoclasts, is a gene that is directly regulated by HIF-2α^24^, we hypothesized that the decreased osteoclastic activity in *Hif-2α*;^fl/fl^*Ctsk*-*Cre* mice was caused by a decrease in TRAF6 levels. However, the molecular mechanism responsible for the increased number of osteoblasts in *Hif-2α*;^fl/fl^*Ctsk*-*Cre* mice remains to be determined, although it may be due to osteoblast-osteoclast interactions.

Estrogen deficiency in postmenopausal females is another main cause of age-associated net bone loss and subsequently leads to osteoporosis^34^. We previously used an animal model of OVX-induced osteoporosis to evaluate the catabolic role of HIF-2α in bone remodeling during estrogen deficiency-associated osteoporosis^24^. Although the molecular mechanisms underlying pathological bone diseases caused by estrogen deficiency and aging differ somewhat, our findings suggest that HIF-2α is a crucial regulator of both estrogen deficiency- and aging-associated osteoporosis. While osteoporosis generally occurs in women, approximately 12% of men aged >50 years old are expected to experience an osteoporotic fracture during their lifetime^53^. Almost 30% of patients with hip fractures are men, and mortality rates within 1 year after fracture are higher in men than in women^54^. These findings suggest that some pathophysiological mechanisms of aging-induced osteoporosis are independent of estrogen deficiency. There are two possible mechanisms of estrogen-independent age-induced osteoporosis: vitamin D deficiency and age-related osteoblast dysfunction^55^. Aging reduces the production of vitamin D in the skin to almost 50% of the normal level and reduces the intestinal concentration of vitamin D receptor, reducing calcium absorption^56^. Animal studies have shown that the ability of MSCs to differentiate into osteoblasts is impaired by aging^57^. Furthermore, the senescence-associated secretory phenotype can cause age-related dysfunction in other tissues. For example, aging and cell senescence in bone can induce dysfunction of osteoblast progenitor cells, induce defective bone formation and increase osteoclastogenesis^11,58^. However, the molecular mechanisms by which senescence affects osteoblasts and disturbs bone homeostasis remain unclear.

HIF-2α may play an important role in senescence-mediated osteoporosis. Although several drugs, such as bisphosphonates and parathyroid hormone, are currently used to treat osteoporosis^59,60^, they are ineffective against osteoporosis that has already developed. In addition, few therapeutic targets have been identified for treating osteoporotic bone loss in elderly individuals. Targeting senescence-related intrinsic factors involved in bone homeostatic dysfunction may contribute to the development of a novel class of antiaging therapies. Our current results suggest that HIF-2α is a key regulator of age-related bone loss, as well as the pathogenesis of osteoporosis. Treatment with a potent HIF-2α inhibitor resulted in the recovery of osteoblast function reduced by doxorubicin-mediated senescence. These findings indicate that inhibition of HIF-2α in aged bones not only results in the reversal of age-related bone loss that has already occurred by restoring osteoblast function but also prevents age-mediated osteoporosis by inhibiting osteoclast differentiation and maturation.

## Supplementary information

Supplemental information
