# Supplementary material for: Hypoxia-inducible factor-2α mediates senescence-associated intrinsic mechanisms of age-related bone loss
Source: Exp Mol Med. 2021 Apr 2;53(4):591–604. doi: 10.1038/s12276-021-00594-y (PMC8102580; doi:10.1038/s12276-021-00594-y)
Supplement: Supplementary file 1 — Supplemental information [file 12276_2021_594_MOESM1_ESM.docx]

**Supplementary Information**

**Hypoxia-inducible factor-2α mediates senescence-associated intrinsic mechanisms of age-related bone loss**

Sun Young Lee^1^, Ka Hyon Park^1^, Gyuseok Lee^1^, Su-Jin Kim^1,2^, Won-Hyun Song^1,2^, Seung-Hee Kwon^1,2^, Jeong-Tae Koh^1,2^, Yun Hyun Huh^3^ and Je-Hwang Ryu^1,2*^

Supplementary Figure 1 to 8

Supplementary Table 1

**Supplementary Figures**

**
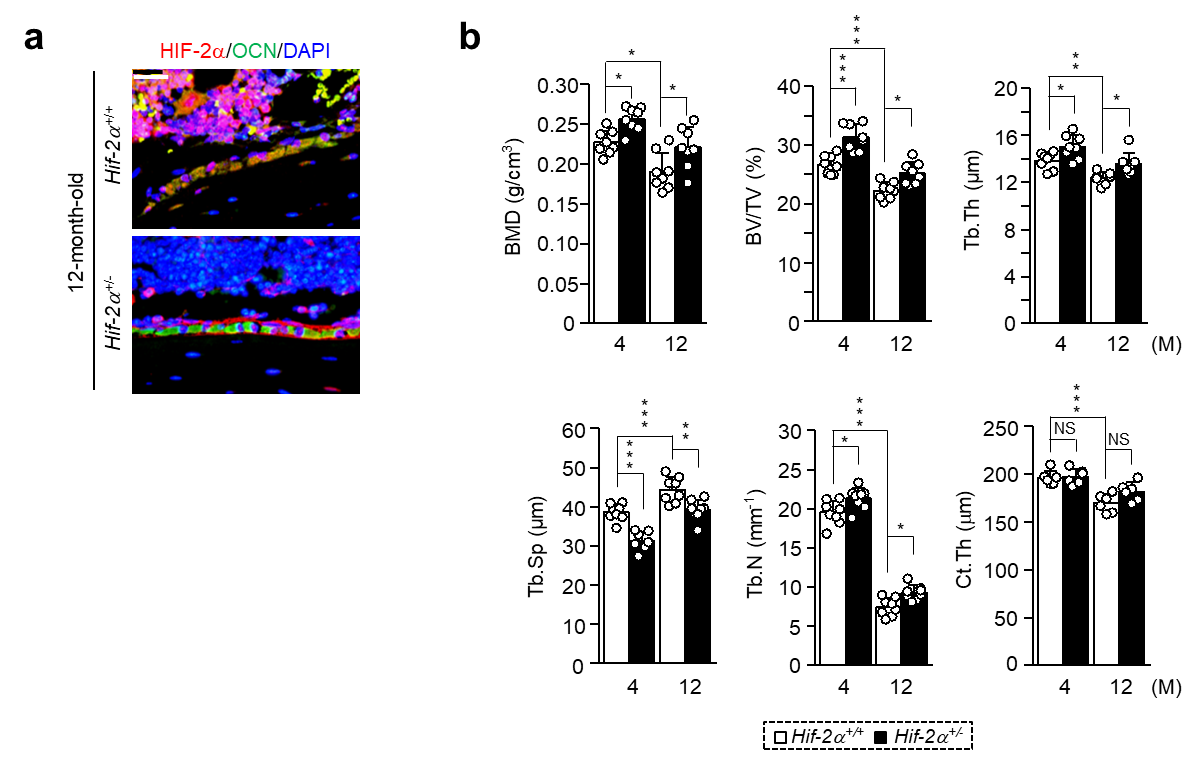
**

**Supplementary Figure 1.** Heterozygous *Hif-2α* KO mice alleviate age-dependent osteoporotic phenotype. (**a**) Co-localization of HIF-2α and OCN was determined by double immunostaining. (n=3; scale bar, 25 μm). Nuclei were detected by DAPI. (**b**) BMD, BV/TV, Tb.Th, Tb.Sp, Tb.N and Ct.Th were assessed using µCT analysis. (M) = Month. Values are presented as means ± SD (**P* < 0.05, ***P* < 0.01, ****P* < 0.005 and NS = not significant).


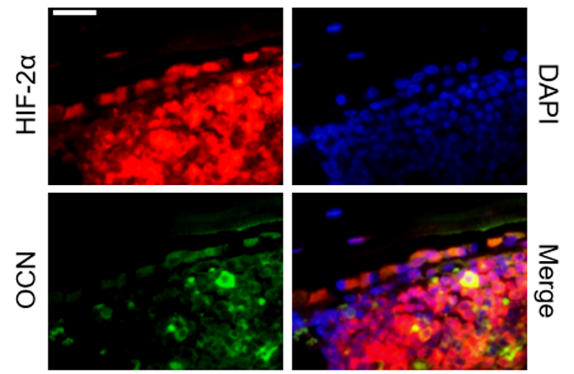


**Supplementary Figure 2.** Double staining of HIF-2α and OCN in osteoblasts of aged mice. OCN was detected in HIF-2α-expressing osteoblasts in 12-month-old bones. Nuclei were detected by DAPI staining. Scale bar: 25 μm.


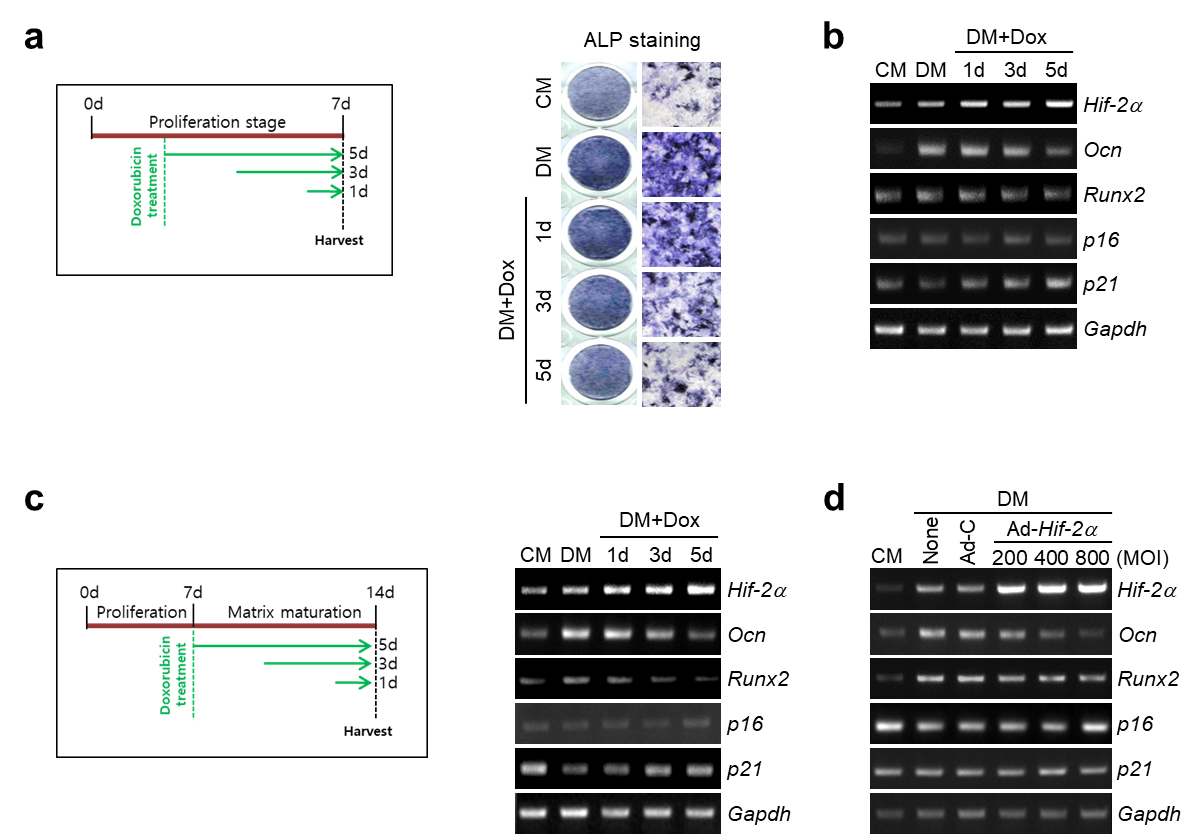


**Supplementary Figure 3.** Doxorubicin inhibits osteoblast differentiation followed by induction of cell senescence. (**a** and **b**) Effect of doxorubicin during the osteoblast proliferation phase. Scheme of the experimental design is shown in the left panel. Primary cultured preosteoblasts were maintained in differentiation medium (DM) containing L-AA and β-Gp for 7 days in the absence or presence of doxorubicin for the indicated periods of time. CM, control media. Level of osteoblast differentiation was assessed by ALP staining (**a**). Levels of *Hif-2α*, *Ocn*, *Runx2*, *p16* and *p21* transcripts were assessed by RT-PCR and normalized against levels of *Gapdh* mRNA in the same samples (n = 5 ; **b**). (**c**) Effect of doxorubicin during the osteoblast matrix maturation process. Under other conditions, doxorubicin induced senescence after 9 days of osteoblast maintenance in differentiation media (DM) containing L-AA and β-Gp or control media (CM) (n = 5). Scheme of the experimental design is shown in the left panel. mRNA levels of the indicated genes were assessed by RT-PCR analyses (right panel). (**d**) Detection of the indicated mRNAs in osteoblasts infected with 800 MOI of Ad-C (control) or the indicated MOI of Ad*-Hif-2α* (n ≥ 4)*.*


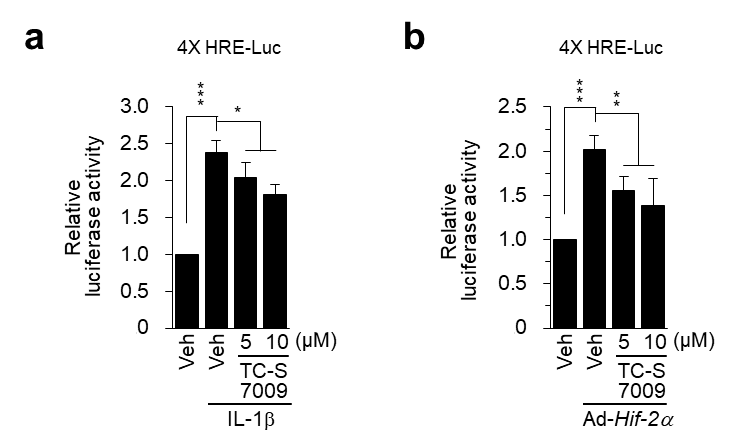


**Supplementary Figure 4.** Verification that TC-S 7009 inhibits HIF-2α activity. (**a**) Primary chondrocytes transfected with 4xHRE-luc and pCMV-β-gal were treated with 2 ng/ml IL-1β in the presence or absence of TC-S 7009 (n = 5). (**b**) Transfected cells were infected with Ad-C or Ad-*Hif-2α* (800 MOI) and treated with or without TC-S 7009 (n = 5). Luciferase activities were normalized with respect to the corresponding β-gal activities. Values are presented as means ± SD (**P* < 0.05, ***P* < 0.01 and ****P* < 0.005).


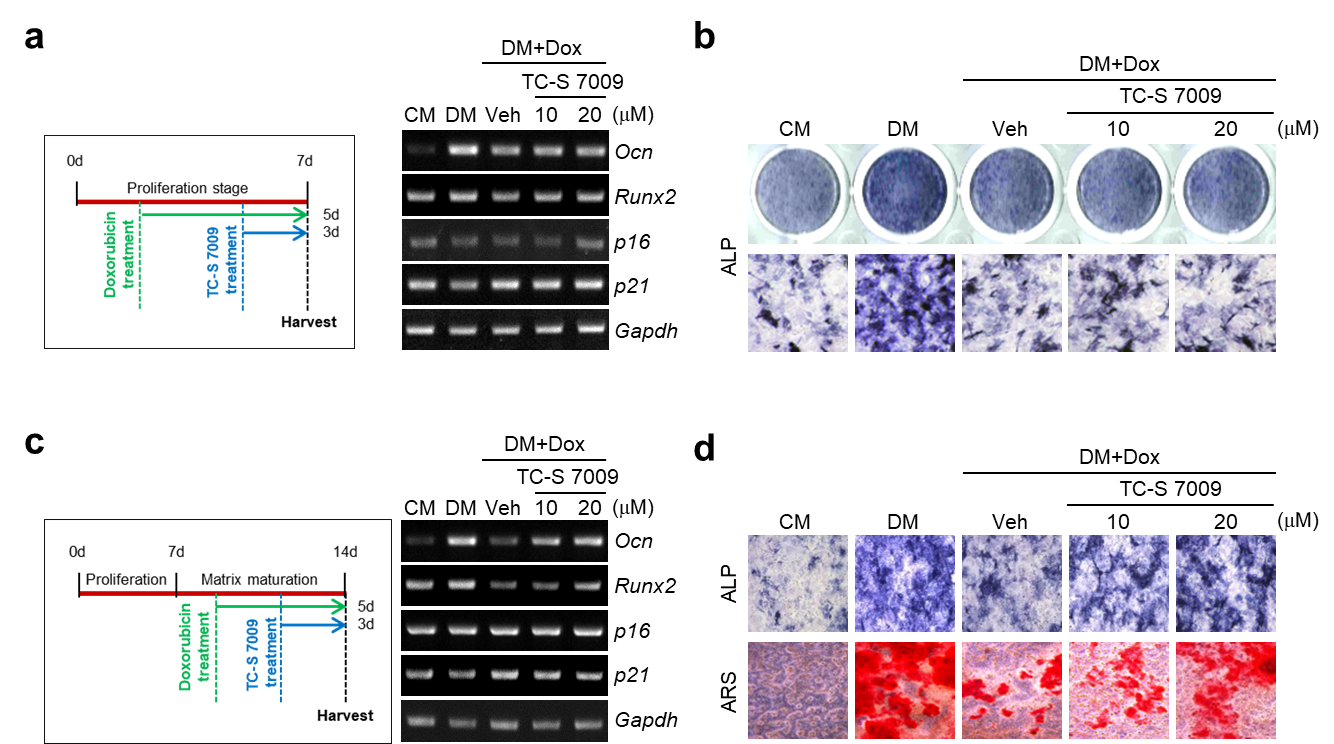


**Supplementary Figure 5.** HIF-2α inhibition by TC-S 7009 blocks doxorubicin-induced impaired osteoblast matrix maturation. (**a** and **b**) Effect of TC-S 7009 on the osteoblast proliferation phase. Schematic diagram of the experimental design is shown in the left panel. Primary cultured preosteoblasts were incubated in differentiation media (DM) containing L-AA and β-Gp for 7 days in the absence or presence of doxorubicin, and exposed to the indicated concentrations of TC-S 7009 on day 4 of differentiation. Transcript levels of *Ocn*, *Runx2*, *p16* and *p21* were examined by RT-PCR (n = 5; **a**) and osteoblast differentiation was visualized by ALP staining (**b**). (**c** and **d)** Effect of TC-S 7009 on the osteoblast matrix maturation phase. Preosteoblsts were stimulated with doxorubicin on day 9 of differentiation and treated TC-S 7009 on differentiation day 11. Transcript levels of *Ocn*, *Runx2*, *p16* and *p21* were examined by RT-PCR (n = 5; **c**). Representative enlarged images of ALP and ARS are presented (**d**).

**
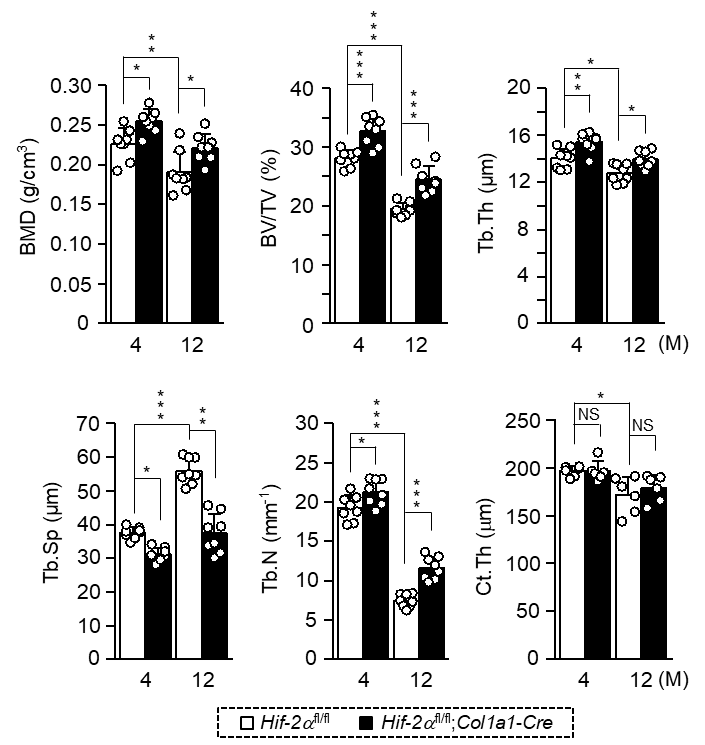
**

**Supplementary Figure 6.** Osteoblast-specific depletion of HIF-2α blocks age-dependent osteoporotic bone loss. BMD, BV/TV, Tb.Th, Tb.Sp, Tb.N and Ct.Th were assessed using µCT analysis. (M) = Month. Values are presented as means ± SD (**P* < 0.05, ***P* < 0.01, ****P* < 0.005 and NS = not significant).

**
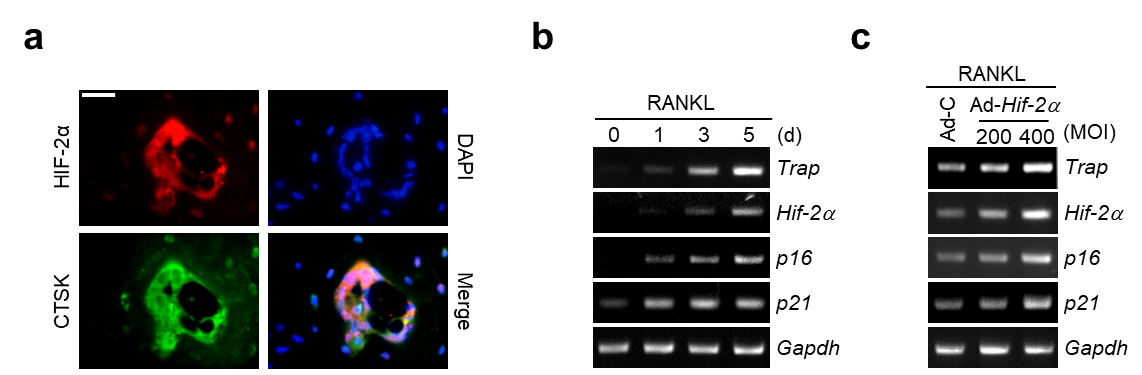
**

**Supplementary Figure 7.** HIF-2α-expressing osteoclasts show increased phenotype of osteoclastogenesis. (**a**) Double staining of HIF-2α and CTSK in osteoclasts of 12-month-old mice. Scale bar : 25 μm. (**b**) RT-PCR analysis of *Trap*, *Hif-2α*, *p16* and *p21* mRNA levels during RANKL-induced osteoclast differentiation (n = 5). (**c**) Levels of *Trap*, *Hif-2α*, *p16* and *p21* mRNAs in osteoclasts infected with Ad-C or Ad-*Hif2α* (n ≥ 5)*.*


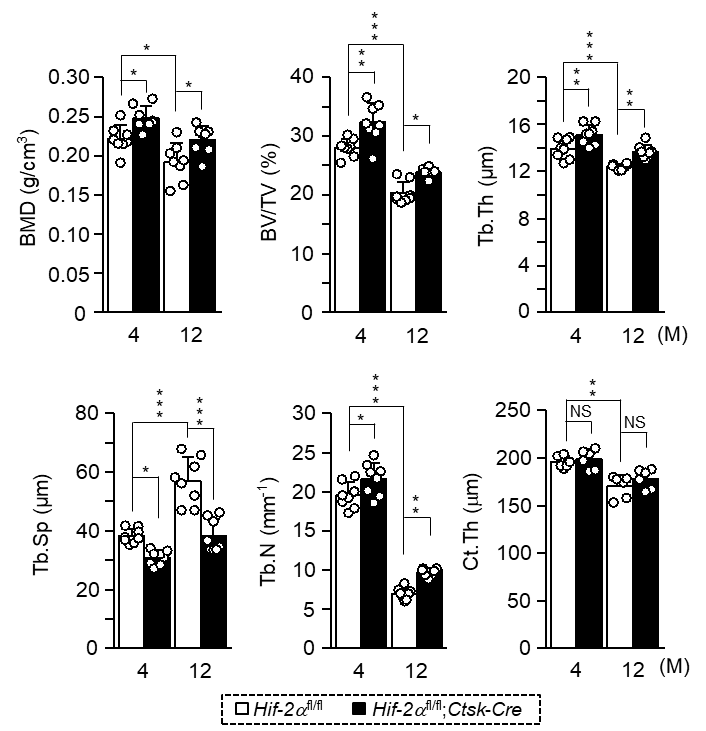


**Supplementary Figure 8.** Osteoclast-specific depletion HIF-2α suppresses bone loss in aged mice. BMD, BV/TV, Tb.Th, Tb.Sp, Tb.N and Ct.Th were assessed using µCT analysis. (M) = Month. Values are presented as means ± SD (**P* < 0.05, ***P* < 0.01, ****P* < 0.005 and NS = not significant).

**Supplementary Table 1.** PCR primers and conditions

| Gene | Strand | Sequences (5’-3’) | Size  (bp) | ^a^AT  (℃) | Origin |
| --- | --- | --- | --- | --- | --- |
| *Hif-2α* | ^b^S | AGAAGAGCAAAGACGTGTCCACCGAG | 347 | 63 | Mo |
|  | ^c^As | GTAGAACTCATAGGCAGAGCGTCCAAG |  |  |  |
| *Ocn* | S | CTCCTGAGAGTCTGACAAAGCCTT | 320 | 55 | Mo |
|  | As | GCTGTGACATCCATTACTTGC |  |  |  |
| *Runx2* | S | GCCACCTTTACCTACACCCC | 363 | 55 | Mo |
|  | As | GACTCATCCATTCTGCCGCT |  |  |  |
| *Trap* | S | CACGATGCCAGCGACAAGAGGTTC | 366 | 58 | Mo |
|  | As | AAACGTAGTCCTCCTTGGCTGCTGC |  |  |  |
| *p16* | S | GCTCAACTACGGTGCAGATTC | 194 | 60 | Mo |
|  | As | GCACGATGTCTTGATGTCCC |  |  |  |
| *p21* | S | AGAACGGTGGAACTTTGACT | 213 | 60 | Mo |
|  | As | GAGTGCAAGACAGCGACAAG |  |  |  |
| *Gapdh* | S | TCACTGCCACCCAGAAGA | 431 | 60 | Mo |
|  | As | TGTAGGCCATGAGGTCCA |  |  |  |
| *p16*  #1 ChIP | S | GGCGCCTCTGGGAAGC | 131 | 60 | Mo |
|  | As | AGTGACCAAGAACCTGCGAC |  |  |  |
| *p16*  #2 ChIP | S | GGCGCCTCTGGGAAGC | 100 | 60 | Mo |
|  | As | CGGCCCATCTTTGCTCCA |  |  |  |
| *p16*  #3 ChIP | S | GCGAAGCGAGCGGGAT | 117 | 60 | Mo |
|  | As | CCAGTCGCGCGGGAAA |  |  |  |
| *p16*  #4 ChIP | S | AATAAACAACGTCCTCGGGCT | 138 | 60 | Mo |
|  | As | GAACAGCGCGCAGAGAAGAG |  |  |  |
| *p21*  #1 ChIP | S | GATGTATGTGGCTCTGCTGGT | 171 | 60 | Mo |
|  | As | CAACCCACTCCTTCACCGAT |  |  |  |
| *p21*  #2 ChIP | S | GTTGGTCTCCATCGGAATAGGT | 101 | 60 | Mo |
|  | As | GCGGGGTCACGTCTGTAATAA |  |  |  |
| *p21*  #3 ChIP | S | GGCTCCTCCAACCATGTTTCT | 153 | 60 | Mo |
|  | As | CTTCCCAGAGCACCACAAAAG |  |  |  |
| *p21*  #4 ChIP | S | CTTGTGTTTTTGAGGGTCTGCT | 187 | 60 | Mo |
|  | As | AGGCCCACACACACATATAGACTC |  |  |  |

^a^AT,annealing temperature; ^b^S,sense primer; ^c^As,antisense primer
